# Improvement of steatotic rat liver function with a defatting cocktail during *ex situ* normothermic machine perfusion is not directly related to liver fat content

**DOI:** 10.1371/journal.pone.0232886

**Published:** 2020-05-12

**Authors:** Siavash Raigani, Cailah Carroll, Stephanie Griffith, Casie Pendexter, Ivy Rosales, Hany Deirawan, Rafic Beydoun, Martin Yarmush, Korkut Uygun, Heidi Yeh

**Affiliations:** 1 Division of Transplant Surgery, Massachusetts General Hospital, Harvard Medical School, Boston, Massachusetts, United States of America; 2 Center for Engineering in Medicine, Massachusetts General Hospital, Harvard Medical School, Boston, Massachusetts, United States of America; 3 Shriners Hospital for Children, Boston, Massachusetts, United States of America; 4 Department of Pathology, Massachusetts General Hospital, Boston, Massachusetts, United States of America; 5 Department of Pathology, Wayne State University School of Medicine, Detroit, Michigan, United States of America; 6 Department of Biomedical Engineering, Rutgers University, Piscataway, New Jersey, United States of America; Medizinische Fakultat der RWTH Aachen, GERMANY

## Abstract

There is a significant organ shortage in the field of liver transplantation, partly due to a high discard rate of steatotic livers from donors. These organs are known to function poorly if transplanted but make up a significant portion of the available pool of donated livers. This study demonstrates the ability to improve the function of steatotic rat livers using a combination of *ex situ* machine perfusion and a “defatting” drug cocktail. After 6 hours of perfusion, defatted livers demonstrated lower perfusate lactate levels and improved bile quality as demonstrated by higher bile bicarbonate and lower bile lactate. Furthermore, defatting was associated with decreased gene expression of pro-inflammatory cytokines and increased expression of enzymes involved in mitochondrial fatty acid oxidation. Rehabilitation of marginal or discarded steatotic livers using machine perfusion and tailored drug therapy can significantly increase the supply of donor livers for transplantation.

## Introduction

Liver transplantation remains the only cure for end-stage liver disease. However, there continues to be a significant organ shortage with approximately 14, 000 patients on the waitlist and 8,000 liver transplants performed annually [1]. Central to the donor organ shortage is the high discard rate of procured or potentially procurable organs, with nearly 3,000 livers discarded per year [2]. As a result of the obesity epidemic in the United States and Europe, nearly half of procured livers are discarded due to excessive macrosteatosis related to non-alcoholic fatty liver disease (NAFLD) [3].

Steatotic livers are particularly prone to ischemia-reperfusion injury (IRI)[4]. As a result, transplantation of livers with moderate (30–60%) or severe (>60%) macrosteatosis is associated with increased early allograft dysfunction (EAD) or primary non-function, and decreased long-term graft survival [5, 6]. Further contributing to IRI is acute-on-chronic oxidative stress, compromised hepatic microcirculation, and an inability to recover from an energy-depleted state [7–10].

The application of *ex situ* machine perfusion technology to liver resuscitation and rehabilitation has not only decreased the organ discard rate, but expanded the ability to use marginal, extended criteria, or even discarded livers for transplantation without compromising patient and graft survival [11, 12]. Using machine perfusion as a platform for targeted intervention paves the way for salvage of otherwise discarded organs for potential transplantation. One application of this technology involves salvaging steatotic livers. Over 10 year ago, our group reported for the first time in the literature a combination of agents capable of “defatting” and reversing key biochemical deficiencies in steatotic hepatocytes in culture [13–15]. Boteon and colleagues recently showed that the same cocktail decreases fat content and improves perfusion parameters in discarded steatotic human livers [16]. However, the safety and utility of several components of the cocktail have not been confirmed or have been abandoned for human clinical use [17]. We therefore performed a mechanistic study in a rat liver perfusion model to identify pathways targeted by these agents, with the ultimate goal of replacing them with agents developed in the interim 10 years that are safe for human clinical use.

## Methods

### Preparation of perfusate

Baseline (plain) perfusate consisted of high-glucose Dulbecco’s Minimum Eagles’ Medium supplemented with 10% v/v fetal bovine serum, 2% v/v penicillin-streptomycin, and 3% w/v bovine serum albumin. The original development of the defatting cocktail *in vitro* [13, 14] and its subsequent cytotoxicity testing in cell culture models [15] have been previously described. Defatting cocktail agents include 10 uM forskolin, 1uM GW7647, 1uM GW501516, 10 uM scoparone, 10 uM hypericin, 0.4 ng/ml visfatin, 0.8 mM L-carnitine, and additional amino acids [13, 14]. Defatting perfusate consisted of plain perfusate supplemented with defatting cocktail. Perfusate and cocktail component details are provided in the Supplemental Methods in S1 File.

### Isolated liver perfusion

Lean (Fa/fa) and obese (fa/fa) male Zucker rats (Charles River Laboratories, Wilmington, MA) aged 12–16 weeks and group housed in pairs in a conventional room under standard conditions (21ºC room temperature, 12-hour light/dark cycles, mixed paper/cellulose bedding). Rats had free access to standard chow and autoclaved water. Animal studies were approved by the institutional animal use and care committee of the Massachusetts General Hospital and Shriners Hospital for Children and comply with the *Guide for Care and Use of Laboratory Animals* as outlined by the National Academy of Sciences. After induction of anesthesia with 3–5% isoflurane, a transverse laparotomy was made. The common bile duct was partially transected, cannulated with 28-gauge PTFE tubing, and secured with 4–0 silk sutures. 0.3 mL of 1000U/mL heparin was injected into the inferior vena cava (IVC) and after 3 minutes of circulation, the hepatic artery was ligated with 7–0 silk. Next, the portal vein was cannulated with a 16-gauge catheter, followed immediately by transection of the IVC. The liver was then flushed slowly with 50 mL of ice-cold 0.9% saline via the portal cannula while the rat was simultaneously euthanized by exsanguination under isoflurane gas anesthesia. Following cold flush, the liver was freed from its ligamentous attachments and transferred to the perfusion basin to be perfused *ex situ*. Total cold ischemic time was less than 10 minutes.

Three groups of six rats were used. 6 livers from lean rats (LL) underwent plain perfusion, acting as positive control. 6 livers from obese rats (SL) underwent plain perfusion as negative controls, and 6 livers from obese rats underwent defatting perfusion (DSL).

The perfusion reservoir contained 500 mL of perfusate. A roller pump system (Masterflex L/S, Cole-Parmer, Vernon Hills, IL) was used to propel perfusate through a combination heat exchanger-oxygenator, exposed to a gas mixture of 1-2L/min of 5% CO_2_/95% O_2_ at 37º Celsius, and a bubble trap, before perfusing the liver via the portal vein cannula. A filter was not used in the circuit. Pump flow rate was titrated to a portal perfusion pressure of 10–12 mm Hg measured via water column. Outflow from the liver IVC was collected in the liver basin and returned to the perfusion reservoir for recirculation. Livers were perfused for 6 hours. Perfusate samples were taken from the liver basin immediately after initiation of perfusion, at 30 minutes, and at each hour. Bile was collected and sampled at 1, 2, 4, and 6 hours. Livers were weighed immediately before and after perfusion. The study flowchart is represented in S1 Fig.

### Histologic evaluation

After 6 hours of perfusion, duplicate biopsies were taken from the left lateral lobe and the right medial lobe. One set was snap-frozen in liquid nitrogen for subsequent analysis and one set was formalin-fixed and paraffin-embedded. The liver tissue was sectioned in 5-micron slices, mounted on glass slides, and stained with hematoxylin-eosin. Oil red staining was also performed on sectioned frozen liver tissue. Histopathologic assessment was carried by two blinded pathologists. An image guide previously reported by Hall et al. was used to increase accuracy and interobserver concordance [18]. Estimated fat proportionate area (FPA) was used to evaluate macrosteatotic changes in the liver by examination at 4x and subsequently confirmed at 20x magnification. Microsteatosis was quantified under 20x magnification. Discordant cases were reviewed collectively, and consensus obtained for the final interpretation reported.

### Perfusate and bile analysis

Perfusate and bile samples were collected as above and stored at -80°C for future analysis. Assays for ketone bodies (MAK134, Millipore-Sigma, Burlington, MA), triglyceride (MAK266, Millipore-Sigma), very low-density lipoprotein (VLDL; MAK045, Millipore-Sigma), aspartate aminotransferase (AST; TR70121, Thermo Fisher, Waltham, MA), alanine aminotransferase (ALT; TR71121, Thermo Fisher), were performed on perfusate samples per the manufacturer’s instructions. Biochemistry and blood gas measurements were performed on bile and perfusate samples using a point-of-care handheld device (iSTAT CG4+ and Chem8+ cartridges, Abbott Laboratories, NJ). Oxygen uptake rate (OUR) was calculated as:

$$OUR=\frac{V[C_{O2,inflow}-C_{O2,outflow}]}{liver\;weight}$$

where V is the perfusion flow rate (mL/min) and C_O2_ is the oxygen concentration. C_O2_ was calculated as 0.0031*P_O2_, where P_O2_ is the partial pressure of oxygen (mmHg) and 0.0031 (mL O_2_/(mmHg*dL)) is the solubility of oxygen in aqueous solutions at 37°C [13].

### Determination of mRNA levels with quantitative RT-PCR

Frozen liver tissue from the left lateral lobe of liver was crushed under liquid nitrogen. Total RNA extraction was performed using the RNeasy Mini Kit per the manufacturer’s instructions (Qiagen, Waltham, MA). Complementary DNA (cDNA) was reverse transcribed and quantitative real-time PCR (RT-PCR) was performed using rat Fatty Liver RT^2^ Profile PCR Array (Qiagen) on an Applied Biosystems ViiA 7 reader. Target gene expression was normalized to that of the SL group. Relative quantification of gene expression was performed using the ΔΔCt method [19] and transformed to represent fold change.

### Targeted metabolite analysis

Frozen tissue biopsies at end-perfusion were pulverized (~25mg) and analyzed for energy cofactors using a targeted multiple reaction monitoring analysis on a 3200 triple quadrupole liquid chromatography-mass spectrometry (QTRAP LC/MS-MS) system (AB Sciex, Foster City, CA), as previously described [20]. Adenosine tri-, di-, and mono- phosphate (ATP, ADP, AMP), nicotinamide adenine nucleotide (NADH/NAD^+^), and nicotinamide adenine nucleotide phosphate (NADPH/NADP^+^) tissue levels were quantified and pertinent redox ratios were calculated. Energy charge was defined as [ATP + ADP*0.5]/[ATP+ADP+AMP].

### Statistical analysis

Independent continuous data were analyzed using one-way analysis of variance (ANOVA). When the ANOVA reached significance, individual *t* tests were performed between groups [21]. Repeated measures data were analyzed using a random intercept mixed model with a categorical effect of time, a categorical effect of group, and the group by time interactions. Data are presented as means ± standard error of the mean (SEM), unless otherwise indicated. A P value of 0.05 was considered significant. All statistical tests were performed using Stata v15 (StataCorp, College Station, TX), and graphics were created using Prism 8 (GraphPad, San Diego, CA).

### Network analysis

Network analysis was performed and visualized in Cytoscape [22] with the ReactomeFIViz application which interfaces with the Reactome Functional Interaction database [23]. The network was created using expression data from all genes examined with quantitative RT-PCR (gene set). Linker genes that shared two or more functional interactions with individual targets from the gene set were included to ensure that none of the genes investigated were excluded from the functional network due to a lack of direct interaction.

## Results

### Steatotic livers demonstrate improved function during defatting perfusion

**Fig 1:**
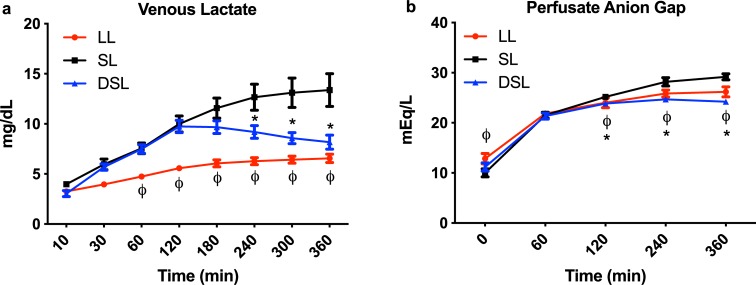
Functional parameters during *ex situ* machine perfusion of Zucker rat livers. (a) Addition of defatting cocktail results in significantly improved lactate clearance and b) anion gap balance during six hours of machine perfusion of steatotic rat livers compared to plain perfusion of steatotic livers. Defatted steatotic livers demonstrate improved anion gap balance compared to control steatotic livers. P<0.05 for random intercept mixed model analysis indicated by * for comparison between DSL and SL, and ϕ for LL and SL groups. LL, control lean livers; SL, control steatotic livers; DSL, defatted steatotic livers.

DSL and SL groups had a similar rise in perfusate lactate content during the first two hours of perfusion, while the LL group had a significantly slower rise as compared to the SL group. After 2 hours of perfusion, DSL livers began clearing lactate, which became significantly lower at hours 4 through 6, compared to the SL group (Fig 1A). Similarly, the anion gap of the perfusate increased initially in all three groups but decreased significantly in the LL and DSL groups starting at the 2^nd^ hour of perfusion compared to the SL group (Fig 1B).

### Defatting perfusion improves the quality of synthesized bile

**Fig 2:**
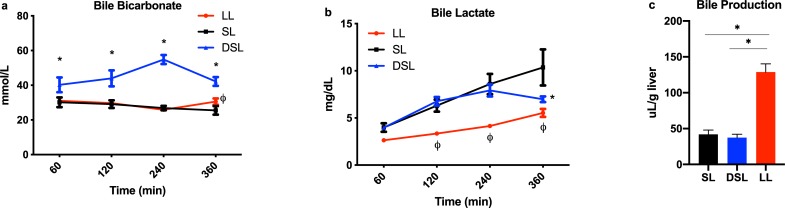
Biomarkers of cholangiocyte health and bile production during liver perfusion. Defatting of steatotic livers (DSL) was associated with significantly improved (a) bicarbonate and (b) lactate levels in collected bile samples compared to control steatotic livers (SL). DSL and SL groups produced similar volumes of bile per gram of liver mass, while the LL group produced significantly higher volumes (c). P<0.05 for random intercept mixed model analysis indicated by * for comparison between DSL and SL, and ϕ for LL and SL groups (a,b); * indicates P<0.05 for *t* tests (c). LL, control lean livers; SL, control steatotic livers; DSL, defatted steatotic livers.

Bicarbonate levels in bile produced by DSL livers were significantly higher compared to the SL group at each measurement point (Fig 2A). Interestingly, DSL livers demonstrated the ability to clear lactate content in collected bile after 6 hours of perfusion compared to persistently rising levels seen in SL livers (Fig 2B). Bile lactate levels in the LL group were notably lower throughout perfusion. DSL and SL groups made similar volumes of bile per gram of liver tissue (37.4 ± 10.5 vs. 42.0 ± 14.8 uL/g, P = 0.58), while LL produced significantly higher volumes (128.7 ± 28.2 uL/g, P<0.001 for both comparisons) (Fig 2C).

### Defatting perfusion associated with decreased pro-inflammatory signaling

**Fig 3:**
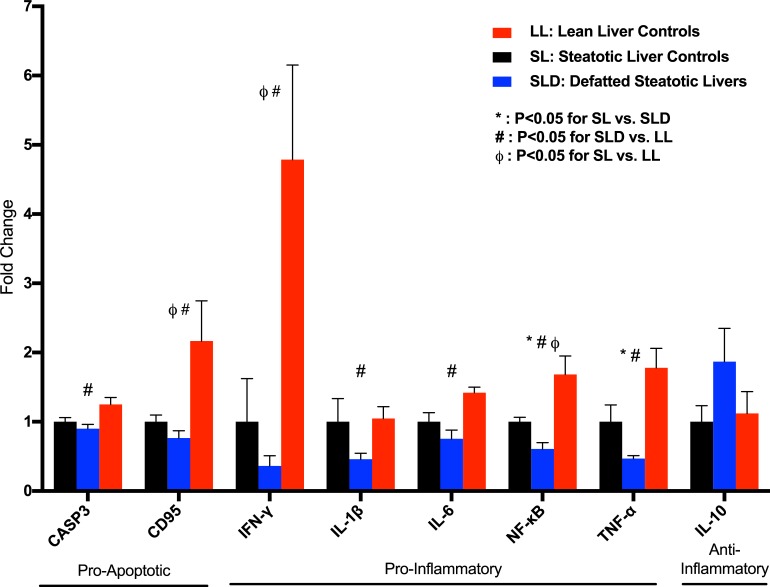
Defatting results in decreased gene expression of pro-inflammatory biomarkers. Defatting of steatotic livers (DSL) results in significantly decreased gene expression of NF-κB and TNF-α compared to both the SL and LL groups. The DSL group also had significantly lower expression of the pro-inflammatory markers IFN-γ, IL-1β, and IL-6, and pro-apoptotic markers caspase-3 and CD95, compared to the LL group. P<0.05 indicated by * for comparison between DSL and SL, # between SLD and LL groups, and ϕ between SL and LL groups. LL, control lean livers; SL, control steatotic livers; DSL, defatted steatotic livers; CASP3, caspase-3; CD95, cluster of differentiation 95; IFN-γ, interferon-gamma; IL-, interleukin-; NF-κB, nuclear factor kappa B; TNF-α, tumor necrosis factor-alpha.

DSL livers demonstrated decreased hepatic expression of inflammatory mediators nuclear factor kappa B (NF-κB; fold change 0.61 ± 0.09, P = 0.005) and tumor necrosis factor-alpha (TNF-α; .47 ± .04, P = 0.025) compared to the SL group (Fig 3). Interleukin-1 beta expression (IL-1β; 0.46 ±.09, P = 0.052) was decreased in DSL livers but did not reach significance compared to the SL group. Interleukin-6 expression was significantly lower compared to LL livers (IL-6; 0.49 ± 0.18, P = 0.004), but not to the SL group (0.75 ± 0.13, P = 0.14). DSL livers had decreased expression of apoptotic markers caspase 3 (CASP3) and Fas cell surface death receptor (FAS/CD95), compared to the LL group only. Interleukin-10 expression, an anti-inflammatory cytokine, was increased in the DSL group but did not reach significance compared to the SL group (IL-10; 1.87 ± 0.48, P = 0.18).

### Defatting perfusion modulates lipid metabolism

**Fig 4:**
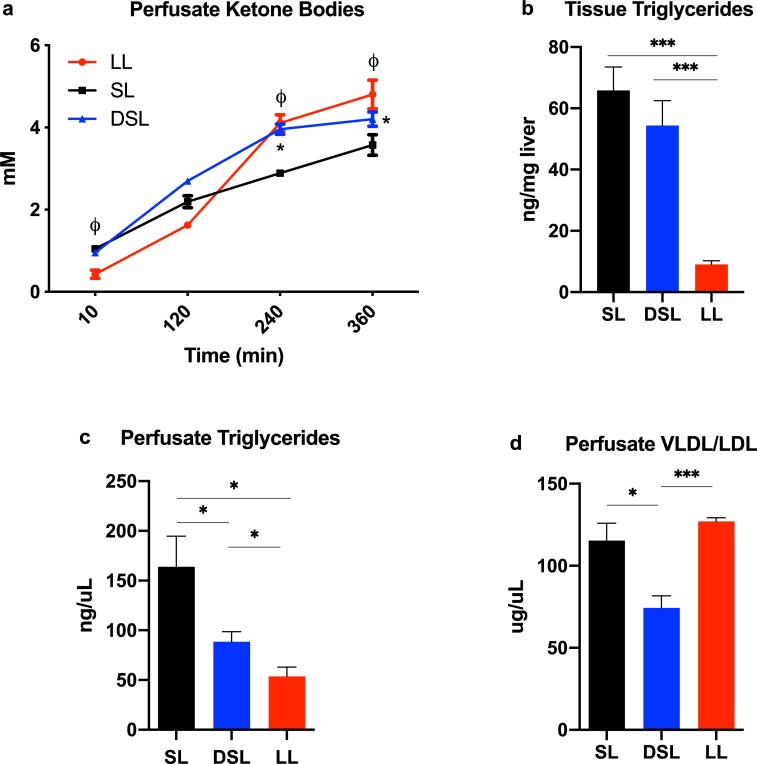
Modulation of lipid metabolism during liver perfusion. (a) Defatting of steatotic livers (DSL) results in significantly increased ketone body synthesis compared to the SL group. (b) Tissue triglyceride levels after six hours of perfusion demonstrate a non-significant decrease in the DSL compared to SL group. (c) Perfusate triglyceride concentrations are significantly higher in the SL group compared to DSL and LL group after six hours of perfusion. This may be the result of cell death as indicated by poor functional parameters in the SL group. (d) The LL group appears to actively synthesize and export very low-density lipoprotein (VLDL), indicating an effective normal function of the perfusion livers in this group. Livers in the DSL group export less VLDL comparatively, indicating either breakdown of VLDL for energy metabolism or a defective cellular process. Perfusate VLDL concentrations in the SL group are comparable to the LL group, though likely as a result of cell death. P<0.05 for random intercept mixed model analysis indicated by * for comparison between DSL and SL, and ϕ for LL and SL groups (a,b); for *t* test comparisons * indicates P<0.05, ** indicates P<0.01, and *** indicated P<0.001. LL, control lean livers; SL, control steatotic livers; DSL, defatted steatotic livers.

The change in perfusate ketone body content, a marker of fatty acid β-oxidation (FAO), was significantly higher at 4 and 6 hours of perfusion in DSL and LL groups compared to SL. Defatting perfusion resulted in a modest decrease in tissue triglyceride content (54.3 ± 8.2 ng/uL) compared to SL livers (65.8 ± 7.7, P = 0.3). Perfusate triglyceride content after 6 hours of perfusion in the DSL group (88.4 ± 10.2 ng/uL) was significantly higher than the LL group (53.7 ± 9.4, P<0.05), but less than the SL group (163.8 ± 30.9, p<0.05). Perfusate content of combined VLDL/LDL after 6 hours of perfusion in the DSL group (74.3 ± 7.4 ug/uL) was significantly lower than LL (127.1 ± 2.2, P<0.001) and SL groups (115.3 ± 10.7, P = 0.01) (Fig 4A–4D).

### Defatting perfusion upregulates fatty acid β-oxidation

**Fig 5:**
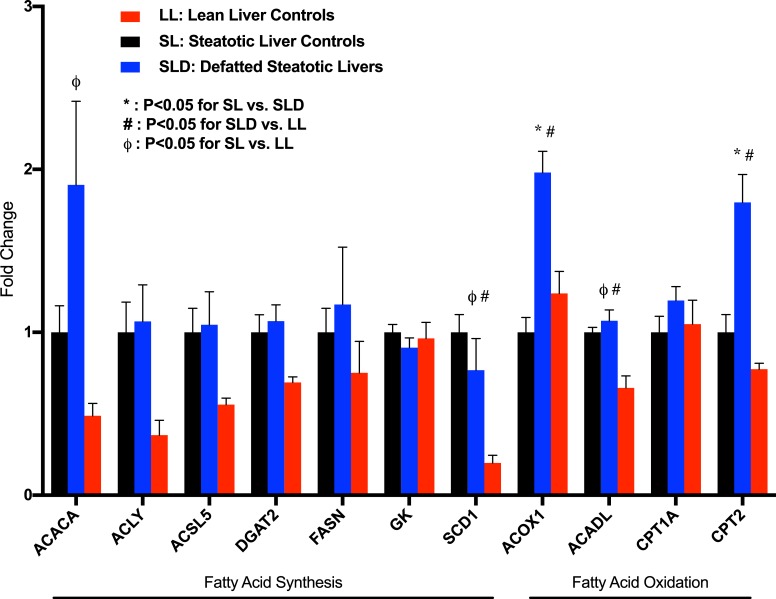
Defatting cocktail upregulated expression of key enzymes involved in mitochondrial fatty acid β-oxidation. Addition of defatting cocktail (DSL) resulted in significantly increase gene expression of acetyl CoA oxidase-1 (ACOX1) and carnitine palmitoyltransferase-2 (CPT2) compared to control steatotic livers (SL). There were no significant differences gene expression of enzymes involved in fatty acid synthesis between DSL and SL groups. Lean livers demonstrated overall decreased expression of enzymes involved in fatty acid synthesis. P<0.05 indicated by * for comparison between DSL and SL, # between SLD and LL groups, and ϕ between SL and LL groups. LL, control lean livers; SL, control steatotic livers; DSL, defatted steatotic livers; ACACA, acetyl-CoA carboxylase alpha; ACLY, ATP citrate lyase; ACSL5, acyl-CoA synthetase long chain family member 5; DGAT2, diacylglycerol O-acyltransferase 2; FASN, fatty acid synthase; GK, glycerol kinase; SCD1, stearoyl-CoA desaturase; ACADL, long chain acyl-CoA dehydrogenase; CTP1a, carnitine palmitoyltransferase 1A.

**Fig 6:**
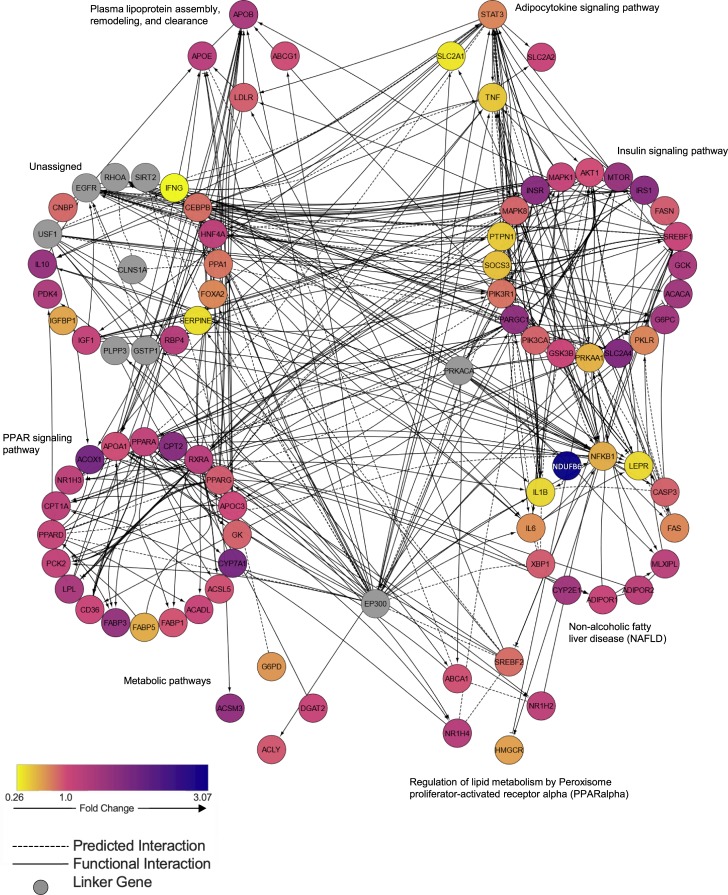
Network analysis of genes involved in metabolism during defatting of steatotic livers. Network analysis of differentially expressed genes after six hours of defatting perfusion in steatotic rat livers relative to control steatotic livers demonstrates upregulated signaling in PPAR-alpha and insulin pathways. EP300, E1A binding protein p300; PRKACA, protein kinase cAMP-activated catalytic subunit alpha; USF1, upstream transcription factor 1; EGFR, epidermal growth factor receptor; RHOA, ras homolog family member A; SIRT2, sirtuin 2; CLNS1A, chloride nucleotide-sensitive channel 1A; GSTP1, glutathione S-transferase pi 1; PLPP3, phospholipid phosphatase 3.

Compared to the SL group, there was significantly increased hepatic gene expression of mitochondrial fatty acid β-oxidation markers acyl-CoA oxidase 1 (ACOX1; fold change 1.98 ± 0.13, P<0.001) and carnitine palmitoyltransferase 2 (CPT2; fold change 1.80 ± 0.17, P = 0.004), though CPT1a and long chain acyl-CoA dehydrogenase (ACADL) expression did not reach significance (Fig 5). SL and DSL groups demonstrated similar hepatic expression of genes associated with fatty acid synthesis (ACACA, ACLY, ACSL5, FASN, SCD1), while the LL group demonstrated lower expression. Apolipoprotein B (APOB) expression was significantly higher in DSL (fold change 1.43 ± 0.14, P = 0.026) and LL groups (1.64 ± 0.14, P = 0.002) compared to SL livers, though expression of apolipoprotein -A1, -C3, and -E (APOA1, APOC3, APOE) was unchanged (S1 Table). Interestingly, lean livers had an unexpectedly higher VLDL/LDL perfusate content, indicating the high synthetic capacity of these livers to produce lipoproteins for transport, further supported by the significantly increased gene expression of HMGCR (fold change 2.85 ± 0.65, P = 0.013 compared to DSL group), the rate-limiting enzyme in the cholesterol synthesis pathway. Fig 6 demonstrates network analysis of genes differentially expressed in DSL compared to SL livers.

### Macrosteatosis decreases with plain and defatting perfusion in rat model

**Fig 7:**
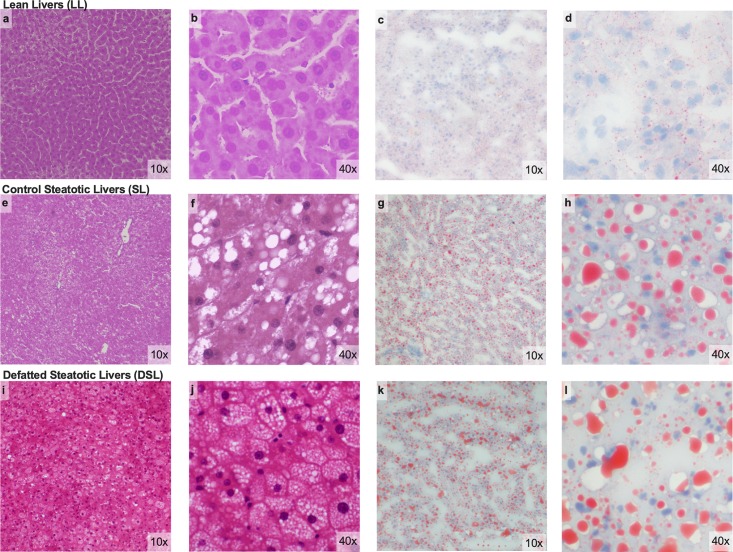
Microscopic assessment of rat liver histology after perfusion. Low and high-grade magnification of H&E and oil red staining performed on rat liver tissue after six hours of perfusion. Lean livers (LL) demonstrate no Macrosteatosis on H&E (a, b) and minimal oil red straining of microdroplets (c,d). Control steatotic livers (SL) demonstrate mild macrosteatosis and variable microsteatosis (e-h). Defatted steatotic livers (DSL) demonstrated mild macrosteatosis and more prevalent microsteatosis (i-l). LL, control lean livers; SL, control steatotic livers; DSL, defatted steatotic livers; H&E, hematoxylin and eosin.

Prior to perfusion, steatotic rat livers were expected to have at least 30% macrosteatotic content, though steatosis varied widely between rats of the same age based on visual inspection. After 6 hours of perfusion, all 6 SL livers had <30% macrosteatosis (range 0–27.5%) versus 5 DSL livers (range 2.5–35%, P = 0.3). Macrosteatosis in the LL group ranged 0–1% after perfusion. Quantified macrosteatotic droplets by oil red staining did not differ between SL (551 ± 181 droplets/mm^2^) and DSL livers (611 ± 125, P = 0.8). Representative histology from each group is shown in Fig 7.

### Defatting perfusion does not improve synthetic function but is associated with hepatoxicity

**Fig 8:**
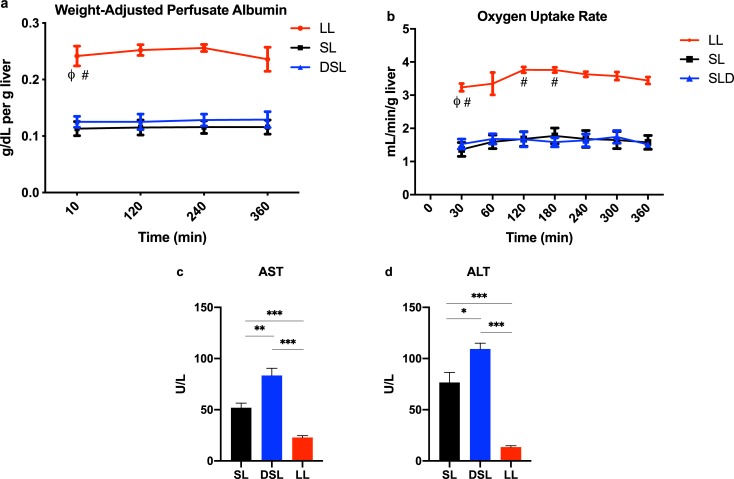
Albumin production and hepatic injury markers after six hours of liver perfusion. (a) Lean livers produced significantly more albumin per gram of liver tissue compared to SL and DSL groups. P<0.05 for random intercept mixed model analysis indicated by ϕ for comparison between LL and SL groups, and # for comparison between LL and DSL groups. (b) Oxygen uptake rate was similar between SL and DSL livers, but significantly higher in LL livers likely as due to the significant liver weight difference. Defatting cocktail was associated with (c) increased aspartate aminotransferase (AST) and (d) alanine aminotransferase (ALT) levels compared to both SL and LL groups. Notably, AST and ALT levels were significantly higher in the SL compared to LL group as well. * indicates P<0.05, ** indicates P<0.01, and *** indicated P<0.001 for *t* tests after analysis of variance indicated significant differences between groups.

Hepatic synthetic function as indicated by the change in weight-adjusted perfusate albumin was significantly higher in the lean livers, but similar between SL and DSL groups (Fig 8A). Similarly, there was no significant difference in the OUR between SL and DSL livers, but both has significantly lower OURs compared to LL livers (Fig 8B). At end-perfusion, AST and ALT levels were significantly higher in the DSL group compared to both SL and LL groups (Fig 8C and 8D).

### Defatting perfusion depletes available pool of energy cofactors

**Fig 9:**
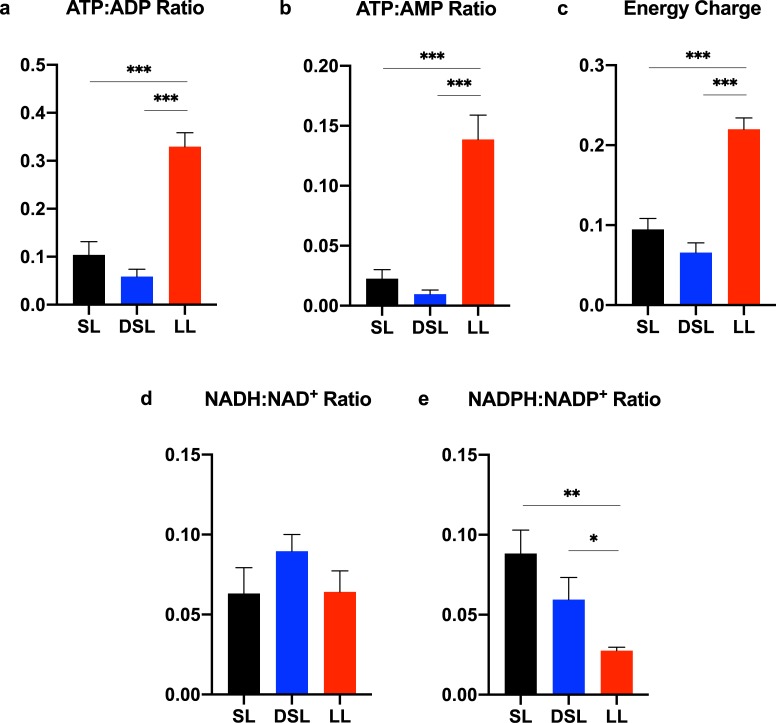
Energy cofactor analysis demonstrates decreased ATP production associated with perfusion of steatotic livers. Control steatotic liver (SL) perfusion results in significantly depleted ATP and energy charge ratios compared to lean livers (LL). Defatting of steatotic livers (DSL) results in similar depletion of energy stores despite evidence of improved function (a-c). NADH:NAD^+^ ratios are similar between the three groups after six hours of perfusion (d), whereas NADPH:NADP^+^ is significantly higher in SL and DSL groups compared to LL (e). Energy charge ratio calculated as [ATP+(ADP*0.5)]/[ATP+ADP+AMP]. indicates P<0.05, ** indicates P<0.01, and *** indicates P<0.001. ATP, adenosine triphosphate; ADP, adenosine diphosphate; AMP, adenosine monophosphate; NADH/NAD^+^, reduced/oxidized nicotinamide adenine dinucleotide; NADPH/NADP^+^, reduced/oxidized nicotinamide adenine dinucleotide phosphate; LL, control lean livers; SL, control steatotic livers; DSL, defatted steatotic livers.

Despite improved functional biomarkers in the DSL group, defatting perfusion resulted in significantly depleted ATP:ADP, ATP:AMP, and energy charge ratios compared to lean livers (Fig 9A–9C). NADH:NAD^+^ ratios were comparable between groups, though NADPH:NADP^+^ ratios were higher in the SL (0.088 ± 0.015) and DSL groups (0.059 ± 0.014) and compared to the LL group (0.028 ± 0.002, P<0.05 for both) (Fig 9D and 9E).

### Steatotic rat livers demonstrate insulin resistance, worsened by defatting perfusion

Perfusate glucose levels increased in both SL and DSL groups during perfusion, but at a significantly higher rate in the DSL group (S2A Fig). The LL group glucose content remained significantly lower throughout perfusion. DSL livers also had significantly increased expression of the gluconeogenic enzymes, glucose-6-phosphatase (G6PC) and pyruvate dehydrogenase kinase 4 (PDK4), compared to both SL and LL groups, which may be an adverse effect of pregnane X receptor (PXR) activation by hypericin. Interestingly, DSL livers had higher insulin receptor (INSR) and insulin receptor substrate 1 (IRS1) expression than both SL and LL groups despite higher perfusate glucose content. Of note, peroxisome proliferator activated receptor (PPAR) -α and–β/δ gene expression was not significantly increased in the DSL group (S1 Table). Finally, during 6 hours of perfusion, LL and SL perfusions had similar perfusion flow rates, while DSL perfusions experienced slightly lower flow rates to maintain similar perfusion pressures (S2B Fig). DSL livers were noted to gain weight significantly compared to SL livers, which lost weight (6.5 ± 3.4 g versus -1.9 ± 1.2, p<0.001).

## Discussion

There is increasing interest in applying machine perfusion technology for rehabilitation of untransplantable livers [24, 25]. Targeting steatotic livers, given their high discard rate, is an ideal application of this platform. A recent proof of concept study by Boteon et al. using the defatting cocktail reported here, demonstrated its effectiveness in improving functional parameters and decreasing macrosteatosis in discarded human steatotic livers [16]. Because of concerns with safety in humans of several components, we performed a mechanistic study in a rat liver perfusion model in order to identify agents safe for human clinical trials with the potential to perform the same functions. We corroborated the prior report of improved functional parameters and bile quality with defatting cocktail but demonstrate notable off-target and adverse effects related to the cocktail components that can be optimized in future studies.

For example, visfatin was thought to be an insulin-mimetic signaling protein, yet the original manuscript describing its action has been retracted [26]. Other studies characterize its function as a pro-inflammatory cytokine found in higher levels with worsening steatosis [27, 28]. Hypericin, a component of St. John’s wort, activates PXR and upregulates enzymes involved in drug metabolism, including cytochrome P450 3A4 (CYP3A4) [29]. PXR activation is associated worsening steatosis and insulin resistance, though it does have anti-inflammatory activity through downregulation of NF-κB target genes [30]. CYP3A4 is also involved in metabolism of numerous drugs, including tacrolimus and cyclosporine, which has implications for post-transplant immunosuppression regimens. Other cocktail components are efficacious at increasing FAO but lack safety data in humans. PPAR-α agonist, GW7647, and PPAR-β/δ agonist, GW501516, demonstrate notably upregulated downstream effects in this study and prior ones, but have never been used in humans. Similarly, forskolin and scoparone are herbal extracts with reported anti-inflammatory and steatosis-reducing effects in animal studies but have not been rigorously tested in humans [30–32]. Carnitine, an over-the-counter supplement, appears to be the only safe and effective component of the cocktail that can be used in future studies [14]. Our understanding of the pathophysiology of NAFLD and NASH has greatly improved in the last decade, and clinicians are now on the verge of a new era were pharmaceutical therapy is becoming available for the treatment of these diseases [33].

The defatting cocktail in this study does demonstrate effectiveness in targeting several pathways. Most remarkable is the downregulation of pro-inflammatory targets TNF-α and NF-κB in defatted compared to untreated livers. The overall anti-inflammatory effect of the defatting cocktail is consistent with reported mechanisms of several components, including activation of PPAR-α and -β/δ (GW7646, GW501516), PXR (hypericin), and constitutive androstane receptor (CAR; scoparone) [30]. Another upregulated pathway involves mitochondrial FAO. We found that ketone body production, a marker of FAO, was significantly higher in the DSL group compared to the SL group. This is further supported by increased expression of CPT2 and ACOX1 genes, key enzymes involved in mitochondrial FAO. Given the safety concerns of the cocktail components, it is worth examining recently-developed drugs under clinical trial that target similar pathways. Elafibranor, a dual PPAR-α/δ agonist, has demonstrated efficacy in decreasing inflammation (via NF-κB targets) and increasing FAO by upregulating expression of CPT1α and ACOX1 [34, 35]. In an international, randomized, double-blind placebo-controlled trial of patients with NASH, elafibranor significantly reduced liver injury enzymes, lipids, glucose profiles, and markers of systemic inflammation. Treatment also resulted in greater resolution of NASH than placebo and was well-tolerated [36]. Other drugs with similar target profiles include pioglitazone, a PPAR-γ agonist that demonstrated improvement in steatosis and lobular inflammation in a phase 3 randomized, placebo-controlled trial of adults with NASH [37]. Another commonly used lipid-lowering agent, bezafibrate, has pan-PPAR agonist activity and reduced hepatic steatosis and gluconeogenesis [38, 39].

One novel finding in this study was that despite increased expression of APOB, DSL livers had lower perfusate VLDL/LDL levels compared to SL livers at end-perfusion. The lower VLDL/LDL levels in the DSL group appears to indicate a process that favors decreased exocytosis of low-density lipoproteins, allowing these molecules to be potentially used for energy metabolism. In the same clinical trial above, elafibranor decreased serum triglyceride and LDL cholesterol levels, increased HDL cholesterol levels, while decreasing liver steatosis, demonstrating potential efficacy for improving the lipid profile and metabolism of steatotic livers during machine perfusion [36].

The farnesoid X receptor (FXR), a nuclear receptor in the same class of the PPARs, has become well-defined in recent years as another metabolic target in the treatment of NASH. The defatting cocktail used in this study did not demonstrate a significant change in gene expression of FXR (S1 Table). In a randomized, placebo-controlled clinical trial, obeticholic acid (OCA; a synthetic FXR agonist) improved steatosis, inflammation, and fibrosis [40], indicating potential efficacy if used in the *ex situ* perfusion setting.

With respect to steatotic liver content, the question as to whether complete defatting is necessary during perfusion to produce a liver viable for transplantation remains unanswered. The lack of a significant difference in macrosteatosis content between SL and DSL groups, despite notable functional improvement, indicates that complete “defatting” or achieving macrosteatosis content below 30% is not the primary outcome of lipid-modulating perfusion. Rather, rehabilitating steatotic livers to function with the characteristics of lean, viable livers should be the goal. This is further corroborated by data from discarded human steatotic livers, which demonstrated improved function and viability in livers that were not defatted to below 30% macrosteatosis after 12 hours of perfusion [16].

There were several limitations to this study. A pre-perfusion liver biopsy was not obtained, making paired comparisons of macrosteatosis and triglyceride content pre- and post-perfusion not possible. Furthermore, defatted livers experienced an unexpected increase in weight during perfusion while plain-perfused steatotic livers had a weight decrease. This was likely due to a portal perfusion pressure that was too high. It is not known why this affected the defatted livers and not the control steatotic livers, though endothelial injury related to the cocktail is a possibility. Another limitation of the rat liver perfusion model is that macrosteatosis decreases in steatotic livers undergoing plain perfusion as well, a finding that is present in the porcine model, but not humans [41, 42]. This may have made any further defatting stimulated by the cocktail difficult to detect by global methods such as histology. It may be that the rat liver perfusion model is exceedingly efficient at inducing metabolism of intracellular lipid droplets or they are being degraded via other mechanisms. Finally, we did not assess *in vivo* function of the control and defatted livers after perfusion using a rat liver transplantation model. This was done in an effort to reduce the number of animals needed while still obtaining novel data to allow further refinement of the drug cocktail prior to scaling up to animal transplantation studies. Future investigations should evaluate the optimized, clinically applicable drug cocktail’s impact on IRI, including mechanisms of complement, ROS release, and liver immune activation in a rat liver transplantation model with improved generalizability, such as diet-induced steatosis, longer cold ischemia, and added warm ischemia.

## Conclusion

Lipid modulation of steatotic rat livers using a drug cocktail that targets mitochondrial fatty acid oxidation is able to improve liver function and bile quality, in addition to decreasing macrosteatosis content. Further research using safe, clinically applicable drugs interventions is required prior to initiating human clinical trials.

## Supporting information

S1 Fig Study flowchart. 6 livers from each group were procured, underwent brief cold ischemia (<10 minutes), and then were normothermically perfused for 6 hours. Perfusate and bile were collected and analyzed at regular intervals. Circulating baseline perfusate was supplemented with the defatting cocktail in the DSL group. Liver tissue was collected for analysis after six hours of perfusion (formalin-fixed and snap-frozen). LL, control lean livers; SL, control steatotic livers; DSL, defatted steatotic livers.
(EPS)

S2 Fig Perfusate glucose content and portal venous flow rates during perfusion of rat livers. (a) Perfusate glucose content during perfusion shows significant variability between groups. Lean livers demonstrate consistent glucose levels, whereas steatotic liver demonstrate rising glucose levels likely as a result of excessive gluconeogenesis. Defatting perfusion appears to worsen the hyperglycemia associated with steatotic livers (b) Portal venous flow rates for a fixed portal venous perfusion pressure of 10–12 mm Hg is similar between SL and LL groups. However, the DSL group demonstrates lower flow rates as a result of edema in the perfusion livers. P<0.05 for random intercept mixed model analysis indicated by * for comparison between DSL and SL, and ϕ for LL and SL groups. LL, control lean livers; SL, control steatotic livers; DSL, defatted steatotic livers.
(EPS)

S1 Table Gene expression of DSL and LL groups relative to SL group. Fold change, standard deviation, P values represented for DSL and LL groups relative to SL group for all genes analyzed using RT-PCR. Numbers in red represent significant values (P<0.05).
(DOCX)

S1 File (DOCX)
