# Supplementary material for: Improvement of steatotic rat liver function with a defatting cocktail during ex situ normothermic machine perfusion is not directly related to liver fat content
Source: PLoS One. 2020 May 12;15(5):e0232886. doi: 10.1371/journal.pone.0232886 (PMC7217452; doi:10.1371/journal.pone.0232886)
Supplement: S1 Table — Fold change, standard deviation, P values represented for DSL and LL groups relative to SL group for all genes analyzed using RT-PCR. Numbers in red represent significant values (P<0.05). (DOCX) [file pone.0232886.s003.docx]

**Supplemental Table 1 – Gene expression of DSL and LL groups relative to SL group**

| Gene (rat) | Gene (human) | Gene, full name | SL  (n=6) | | DSL (relative to SL)  (n=6) | | | LL (relative to SL)  (n=4) | | |
| --- | --- | --- | --- | --- | --- | --- | --- | --- | --- | --- |
|  |  |  | FC | SD | FC | SD | P | FC | SD | P |
| Abca1 | ABCA1 | ATP-binding cassette, subfamily A, member 1 | 1.00 | 0.19 | 0.97 | 0.12 | 0.647 | 1.29 | 0.34 | 0.132 |
| Abcg1 | ABCG1 | ATP-binding cassette, subfamily G, member 1 | 1.00 | 0.33 | 0.98 | 0.18 | 0.678 | 1.23 | 0.40 | 0.447 |
| Acaca | ACACA | Acetyl CoA carboxylase alpha | 1.00 | 0.40 | 1.90 | 1.26 | 0.144 | **0.49** | **0.15** | **0.029** |
| Acadl | ACADL, LCAD | Long chain acyl-CoA dehydrogenase | 1.00 | 0.07 | 1.07 | 0.16 | 0.371 | **0.66** | **0.15** | **0.0011** |
| Acly | ACLY | ATP citrate lyase | 1.00 | 0.45 | 1.07 | 0.55 | 0.979 | **0.37** | **0.18** | **0.019** |
| Acox1 | ACOX1 | Acetyl CoA oxidase 1 | 1.00 | 0.22 | **1.98** | **0.32** | **<0.001** | 1.24 | 0.27 | 0.201 |
| Acsl5 | ACSL5 | Acyl-CoA synthetase long-chain family member 5 | 1.00 | 0.36 | 1.05 | 0.50 | 0.996 | **0.56** | **0.08** | **0.030** |
| Acsm3 | ACSM3 | Acyl-CoA synthetase medium-chain family member 5 | 1.00 | 0.98 | 1.70 | 0.46 | 0.317 | **3.31** | **1.04** | **0.012** |
| Adipor1 | ADIPOR1 | Adiponectin receptor 1 | 1.00 | 0.09 | 1.05 | 0.11 | 0.411 | **0.85** | **0.10** | **0.039** |
| Adipor2 | ADIPOR2 | Adiponectin receptor 2 | 1.00 | 0.22 | 1.20 | 0.26 | 0.225 | 1.51 | 0.53 | 0.076 |
| Akt1 | AKT1 | AKT serine/threonine kinase 1 | 1.00 | 0.18 | 1.00 | 0.26 | 0.929 | **0.61** | **0.11** | **0.004** |
| Apoa1 | APOA1 | Apolipoprotein A1 | 1.00 | 0.28 | 1.06 | 0.59 | 0.918 | **0.55** | **0.10** | **0.011** |
| Apob | APOB | Apolipoprotein B | 1.00 | 0.17 | **1.43** | **0.35** | **0.026** | **1.64** | **0.29** | **0.002** |
| Apoc3 | APOC3 | Apolipoprotein C3 | 1.00 | 0.21 | 1.06 | 0.39 | 0.835 | 0.86 | 0.13 | 0.239 |
| Apoe | APOE | Apolipoprotein E | 1.00 | 0.23 | 1.24 | 0.28 | 0.159 | 1.23 | 0.23 | 0.192 |
| Atp5c1 | ATP5C1 | ATP synthase, H+ transporting, mitochondrial F1 complex, gamma polypeptide 1 | 1.00 | 0.14 | 0.97 | 0.11 | 0.587 | 0.83 | 0.09 | 0.058 |
| Casp3 | CASP3 | Caspase 3 | 1.00 | 0.15 | 0.90 | 0.15 | 0.237 | 1.25 | 0.20 | 0.060 |
| Cd36 | CD36 | CD 36 Molecule | 1.00 | 0.20 | 1.06 | 0.26 | 0.746 | 0.72 | 0.48 | 0.203 |
| Cebpb | CEBPB | CCAAT enhancer binding protein beta | 1.00 | 0.91 | 0.92 | 0.41 | 0.255 | 2.57 | 1.62 | 0.180 |
| Cnbp | CNBP | CCHC-type zinc finger nucleic acid binding protein | 1.00 | 0.08 | 0.87 | 0.12 | 0.053 | **1.21** | **0.15** | **0.025** |
| Cpt1a | CPT1A | Carnitine palmitoyltransferase 1A | 1.00 | 0.24 | 1.20 | 0.21 | 0.211 | 1.05 | 0.29 | 0.879 |
| Cpt2 | CPT2 | Carnitine palmitoyltransferase 2 | 1.00 | 0.27 | **1.80** | **0.42** | **0.004** | 0.77 | 0.07 | 0.109 |
| Cyp2e1 | CYP2E1 | Cytochrome P450, family 2, subfamily E, polypeptide 1 | 1.00 | 0.24 | **1.51** | **0.41** | **0.030** | **1.77** | **0.52** | **0.014** |
| Cyp7a1 | CYP7A1 | Cytochrome P450, family 7, subfamily A, polypeptide 1 | 1.00 | 2.04 | 2.46 | 1.75 | 0.438 | **4.28** | **1.09** | **0.043** |
| Dgat2 | DGAT2 | Diacylglycerol O-acyltransferase 2 | 1.00 | 0.26 | 1.07 | 0.24 | 0.776 | **0.69** | **0.07** | **0.041** |
| Fabp1 | FABP1 | Fatty acid binding protein 1, liver | 1.00 | 0.24 | 0.97 | 0.27 | 0.722 | 0.96 | 0.10 | 0.588 |
| Fabp3 | FABP3 | Fatty acid binding protein 3, muscle and heart | 1.00 | 0.73 | 1.70 | 0.54 | 0.184 | 1.20 | 0.76 | 0.964 |
| Fabp5 | FABP5 | Fatty acid binding protein 5, epidermal | 1.00 | 0.34 | **0.59** | **0.13** | **0.011** | 0.91 | 0.04 | 0.457 |
| Fas | FAS, CD95 | Fas cell surface death receptor | 1.00 | 0.24 | 0.77 | 0.26 | 0.094 | **2.17** | **1.16** | **0.043** |
| Fasn | FASN | Fatty acid synthase | 1.00 | 0.36 | 1.17 | 0.86 | 0.777 | 0.75 | 0.39 | 0.233 |
| Foxa2 | FOXA2 | Forkhead box A2 | 1.00 | 0.24 | 0.76 | 0.21 | 0.072 | 0.89 | 0.16 | 0.350 |
| G6pc | G6PC | Glucose-6-phosphatase, catalytic subunit | 1.00 | 0.32 | **1.50** | **0.30** | **0.030** | 0.99 | 0.26 | 0.793 |
| G6pd | G6PD | Glucose-6-phosphate dehydrogenase | 1.00 | 0.65 | 0.82 | 0.61 | 0.417 | **0.25** | **0.03** | **0.029** |
| Gck | GCK | Glucokinase | 1.00 | 0.29 | 1.74 | 0.91 | 0.099 | 0.64 | 0.22 | 0.054 |
| Gk | GK | Glycerol kinase | 1.00 | 0.12 | 0.91 | 0.15 | 0.225 | 0.96 | 0.20 | 0.673 |
| Gsk3b | GSK3B | Glycogen synthase kinase 3 beta | 1.00 | 0.23 | 1.04 | 0.13 | 0.873 | 1.06 | 0.26 | 0.815 |
| Hmgcr | HMGCR | Hydroxymethylglutaryl-CoA reductase | 1.00 | 0.31 | 0.67 | 0.28 | 0.050 | 1.78 | 0.81 | 0.072 |
| Hnf4a | HNF4A | Hepatocyte nuclear factor 4, alpha | 1.00 | 0.29 | 1.20 | 0.31 | 0.359 | 0.76 | 0.07 | 0.111 |
| Ifng | IFNG | Intergeron gamma | 1.00 | 1.53 | 0.36 | 0.36 | 0.094 | **4.79** | **2.73** | **0.041** |
| Igf1 | IGF1 | Insulin-like growth factor 1 | 1.00 | 0.24 | 1.13 | 0.26 | 0.480 | 1.31 | 0.31 | 0.140 |
| Igfbp1 | IGFBP1 | Insulin-like growth factor binding protein 1 | 1.00 | 0.33 | **0.62** | **0.13** | **0.016** | 1.32 | 0.24 | 0.193 |
| Il10 | IL-10 | Interleukin 10 | 1.00 | 0.57 | 1.87 | 1.18 | 0.180 | 1.12 | 0.63 | 0.953 |
| Il1b | IL-1B | Interleukin 1 beta | 1.00 | 0.82 | 0.46 | 0.21 | 0.052 | 1.05 | 0.34 | 0.703 |
| Il6 | IL-6 | Interleukin 6 | 1.00 | 0.32 | 0.75 | 0.31 | 0.140 | 1.42 | 0.16 | 0.066 |
| Insr | INSR | Insulin receptor | 1.00 | 0.25 | **1.79** | **0.42** | **0.003** | 1.08 | 0.32 | 0.771 |
| Irs1 | IRS1 | Insulin receptor substrate 1 | 1.00 | 0.30 | **1.78** | **0.30** | **0.002** | 1.32 | 0.29 | 0.196 |
| Ldlr | LDLR | Low density lipoprotein receptor | 1.00 | 0.18 | 0.93 | 0.27 | 0.542 | 1.23 | 0.56 | 0.402 |
| Lepr | LEPR | Leptin receptor | 1.00 | 0.89 | 0.40 | 0.14 | 0.055 | 1.36 | 1.07 | 0.803 |
| Lpl | LPL | Lipoprotein lipase | 1.00 | 0.51 | 1.49 | 0.55 | 0.228 | 1.19 | 0.40 | 0.769 |
| Mapk1 | MAPK1, ERK | Mitogen-activated protein kinase 1 | 1.00 | 0.18 | 1.08 | 0.09 | 0.395 | 1.06 | 0.14 | 0.636 |
| Mapk8 | MAPK8, JNK | Mitogen-activated protein kinase 8 | 1.00 | 0.21 | 0.89 | 0.17 | 0.253 | 1.22 | 0.14 | 0.126 |
| Mlxipl | MXLIPL | MAX-like protein X | 1.00 | 0.45 | 1.26 | 0.42 | 0.504 | 0.70 | 0.15 | 0.142 |
| Mtor | MTOR | Mechanistic target of rapamycin (serine/threonine kinase) | 1.00 | 0.22 | **1.65** | **0.55** | **0.026** | 0.75 | 0.15 | 0.068 |
| Ndufb6 | NDUFB6 | NADH:ubiquinone oxidoreductase subunit B6 | 1.00 | 1.91 | 3.36 | 1.27 | 0.101 | 2.76 | 1.06 | 0.332 |
| Nfkb1 | NFKB1, NFKB, p50 | nuclear factor kappa B subunit 1 | 1.00 | 0.16 | **0.61** | **0.22** | **0.005** | **1.68** | **0.54** | **0.019** |
| Nr1h2 | NR1H2, LXRB | nuclear receptor subfamily 1 group H member 2; liver X receptor beta | 1.00 | 0.22 | 1.18 | 0.26 | 0.296 | 1.04 | 0.08 | 0.881 |
| Nr1h3 | NR1H3, LXRA | nuclear receptor subfamily 1 group H member 3; liver X receptor alpha | 1.00 | 0.22 | 1.19 | 0.29 | 0.283 | 1.31 | 0.16 | 0.055 |
| Nr1h4 | NR1H4, FXR | nuclear receptor subfamily 1 group H member 4; farnesoid X receptor | 1.00 | 0.25 | 1.27 | 0.25 | 0.119 | 0.97 | 0.16 | 0.687 |
| Pck2 | PCK2, PEPCK | Phosphoenolpyruvate carboxykinase | 1.00 | 0.28 | 1.11 | 0.20 | 0.573 | 0.85 | 0.08 | 0.244 |
| Pdk4 | PDK4 | Pyruvate dehydrogenase kinase 4 | 1.00 | 0.29 | **1.35** | **0.19** | **0.049** | 0.66 | 0.23 | 0.066 |
| Pik3ca | PIK3CA | Phosphatidylinositol-4,5-bisphosphate 3-kinase catalytic subunit alpha | 1.00 | 0.17 | 0.88 | 0.05 | 0.101 | 1.16 | 0.23 | 0.252 |
| Pik3r1 | PIK3R1 | Phosphoinositide-3-kinase regulatory subunit 1 | 1.00 | 0.18 | **0.82** | **0.07** | **0.034** | **1.45** | **0.31** | **0.021** |
| Pklr | PKLR | Pyruvate kinase, liver and red blood cell isozyme | 1.00 | 0.27 | 0.78 | 0.29 | 0.155 | 0.70 | 0.19 | 0.071 |
| Ppa1 | PPA1 | Pyrophosphatase (inorganic) 1 | 1.00 | 0.14 | **0.82** | **0.13** | **0.030** | 1.25 | 0.21 | 0.058 |
| Ppara | PPARA, NR1C1 | Peroxisome proliferator activated receptor alpha | 1.00 | 0.21 | 1.19 | 0.41 | 0.381 | 0.77 | 0.25 | 0.134 |
| Ppard | PPARD, NR1C2 | Peroxisome proliferator activated receptor delta | 1.00 | 0.44 | 1.11 | 0.29 | 0.901 | 1.02 | 0.28 | 0.818 |
| Pparg | PPARG, NR1C3 | Peroxisome proliferator activated receptor gamma | 1.00 | 0.47 | 1.05 | 0.50 | 0.874 | 1.43 | 0.51 | 0.311 |
| Ppargc1a | PPARGC1A, PGC1A | PPARG coactivator 1 alpha | 1.00 | 0.56 | 1.74 | 0.58 | 0.082 | 0.85 | 0.40 | 0.463 |
| Prkaa1 | PRKAA1, AMPK | Protein kinase AMP-activated catalytic subunit alpha 1 | 1.00 | 0.15 | **0.55** | **0.09** | **<0.0001** | 1.09 | 0.19 | 0.481 |
| Ptpn1 | PTPN1 | Protein tyrosine phosphatase non-receptor type 1 | 1.00 | 0.18 | **0.45** | **0.07** | **<0.0001** | 1.42 | 0.44 | 0.071 |
| Rbp4 | RBP4 | Retinol-binding protein 4 | 1.00 | 0.24 | 1.27 | 0.20 | 0.084 | 1.15 | 0.13 | 0.355 |
| Rxra | RXRA | Retinoid X receptor alpha | 1.00 | 0.26 | 1.27 | 0.39 | 0.234 | 0.92 | 0.27 | 0.559 |
| Scd1 | SCD1 | Stearoyl-CoA destaturase | 1.00 | 0.27 | 0.77 | 0.48 | 0.272 | **0.20** | **0.09** | **<0.001** |
| Serpine1 | SERPINE1, PAI-1 | Serpin family E member 1; plasminogen activator inhibitor 1 | 1.00 | 0.40 | **0.40** | **0.21** | **0.005** | 0.67 | 0.21 | 0.105 |
| Slc27a5 | SLC27A5, ACSB | Solute carrier family 27 (fatty acid transporter), member 5 | 1.00 | 4.03 | 2.05 | 2.16 | 0.844 | 1.19 | 0.48 | 0.568 |
| Slc2a1 | SLC2A1, GLUT1 | Solute carrier family 2 (facilitated glucose transporter), member 1 | 1.00 | 0.48 | **0.36** | **0.17** | **0.006** | 0.86 | 0.19 | 0.407 |
| Slc2a2 | SLC2A2, GLUT2 | Solute carrier family 2 (facilitated glucose transporter), member 2 | 1.00 | 0.12 | 1.06 | 0.24 | 0.619 | 0.95 | 0.27 | 0.662 |
| Slc2a4 | SLC2A4, GLUT4 | Solute carrier family 2 (facilitated glucose transporter), member 4 | 1.00 | 0.36 | 2.70 | 2.50 | 0.143 | **0.50** | **0.03** | **0.015** |
| Socs3 | SOCS3 | Suppressor of cytokine signaling 3 | 1.00 | 0.24 | **0.57** | **0.33** | **0.021** | 1.24 | 0.10 | 0.131 |
| Srebf1 | SREBF1 | Sterol regulatory element binding transcription factor 1 | 1.00 | 0.79 | 1.21 | 0.75 | 0.931 | 0.59 | 0.23 | 0.191 |
| Srebf2 | SREBF2 | Sterol regulatory element binding transcription factor 2 | 1.00 | 0.32 | 0.89 | 0.30 | 0.437 | 0.85 | 0.15 | 0.305 |
| Stat3 | STAT3 | Signal transducer and activator of transcription 3 | 1.00 | 0.24 | 0.75 | 0.18 | 0.052 | 1.04 | 0.26 | 0.895 |
| Tnf | TNF, TNFA | Tumor necrosis factor | 1.00 | 0.59 | **0.47** | **0.11** | **0.025** | 1.78 | 0.56 | 0.115 |
| Xbp1 | XBP1 | X-box binding protein 1 | 1.00 | 0.20 | 0.92 | 0.13 | 0.347 | 1.05 | 0.38 | 0.850 |

FC, fold change; SD, standard deviation; SL, control steatotic livers; DSL, defatted steatotic livers; LL, control lean livers
