# Supplementary material for: Improvement of steatotic rat liver function with a defatting cocktail during ex situ normothermic machine perfusion is not directly related to liver fat content
Source: PLoS One. 2020 May 12;15(5):e0232886. doi: 10.1371/journal.pone.0232886 (PMC7217452; doi:10.1371/journal.pone.0232886)
Supplement: S1 File — (DOCX) [file pone.0232886.s004.docx]

Improvement of steatotic rat liver function with a defatting cocktail during *ex situ* normothermic machine perfusion is not directly related to liver fat content

Siavash Raigani^1,2,3^, Cailah Carroll^2,3^, Stephanie Griffith^2,3^, Casie Pendexter^2,3^, Ivy Rosales^4^, Hany Deirawan^5^, Rafic Beydoun^5^, Martin Yarmush^2,3,6^, Korkut Uygun^1,2,3^, Heidi Yeh^1,2,3^*

^1^ Division of Transplant Surgery, Massachusetts General Hospital, Harvard Medical School, Boston, Massachusetts, USA

^2^ Center for Engineering in Medicine, Massachusetts General Hospital, Harvard Medical School, Boston, Massachusetts, USA

^3^ Shriners Hospital for Children, Boston, Massachusetts, USA

^4^ Department of Pathology, Massachusetts General Hospital, Boston, Massachusetts, USA

^5^ Department of Pathology, Wayne State University School of Medicine, Detroit, Michigan, USA

^6^ Department of Biomedical Engineering, Rutgers University, Piscataway, New Jersey, USA

*Corresponding Author:

[hyeh@partners.org](mailto:hyeh@partners.org) (HY)

**Supplemental Methods**

**Perfusate components, concentration, source, and product numbers**

| **Component** | **Concentration** | **Source** | **Product Number** |
| --- | --- | --- | --- |
| Dulbecco’s Minimum Eagles’ Medium, high glucose | 500 mL | Gibco | 12800082 |
| Fetal bovine serum | 10% v/v | Thermo Fisher | 10437028 |
| Bovine serum albumin | 3% w/v | Millipore Sigma | A7906 |
| Penicillin-streptomycin | 2% v/v | Invitrogen | 15140163 |

**Defatting cocktail components concentration, source, and product numbers**

| **Component** | **Concentration** | **Source** | **Product Number** |
| --- | --- | --- | --- |
| L-Arginine hydrochloride | 252.8 mg/L | Millipore Sigma | 181003 |
| L-Cystine | 48 mg/L | Millipore Sigma | 30200 |
| L-Histidine hydrochloride-H2O | 84 mg/L | Millipore Sigma | 53370 |
| L-Isoleucine | 104.8 mg/L | Millipore Sigma | 4160 |
| L-Leucine | 104.8 mg/L | Millipore Sigma | 4330 |
| L-Lysine hydrochloride | 145 mg/L | Millipore Sigma | 4400-M |
| L-Methionine | 30.2 mg/L | Millipore Sigma | 4500-M |
| L-Phenylalanine | 66 mg/L | Millipore Sigma | 5202 |
| L-Threonine | 95.2 mg/L | Millipore Sigma | 89179 |
| L-Tryptophan | 20.4 mg/L | Millipore Sigma | 6540-M |
| L-Tyrosine | 72 mg/L | Millipore Sigma | 93829 |
| L-Valine | 93.6 mg/L | Millipore Sigma | 94619 |
| Glycine | 30 mg/L | Millipore Sigma | 3570 |
| L-Alanine | 35.6 mg/L | Millipore Sigma | 1250 |
| L-Asparagine | 52.8 mg/L | Millipore Sigma | A4284 |
| L-Aspartic acid | 53.2 mg/L | Millipore Sigma | 11189 |
| L-Glutamic Acid | 58.8 mg/L | Millipore Sigma | 49449 |
| L-Proline | 46 mg/L | Millipore Sigma | 5370 |
| L-Serine | 42 mg/L | Millipore Sigma | 5650 |
| L-Glutamine | 584 mg/L | Millipore Sigma | 49419 |
| Forskolin | 10 uM | Millipore Sigma | F6886 |
| GW7647 | 1 uM | Millipore Sigma | 370698 |
| Scoparone | 10 uM | Millipore Sigma | 254886 |
| GW501516 | 1 uM | Millipore Sigma | SML1491 |
| Hypericin | 10 uM | Millipore Sigma | 400076-M |
| Visfatin | 0.4 ug/L | Millipore Sigma | 68373 |
| L-carnitine hydrochloride | 0.8mM | Millipore Sigma | C0283 |
